# Cisplatin-induced synthetic lethality to arginine-starvation therapy by transcriptional suppression of ASS1 is regulated by DEC1, HIF-1α, and c-Myc transcription network and is independent of *ASS1* promoter DNA methylation

**DOI:** 10.18632/oncotarget.12308

**Published:** 2016-09-28

**Authors:** Yan Long, Wen-Bin Tsai, Jeffrey T. Chang, Marcos Estecio, Medhi Wangpaichitr, Naramol Savaraj, Lynn G. Feun, Helen H.W. Chen, Macus Tien Kuo

**Affiliations:** ^1^ Department of Translational Molecular Pathology, The University of Texas MD Anderson Cancer Center, Houston, Texas, USA; ^2^ Department of Integrative Biology and Pharmacology, University of Texas Health Science Center at Houston, Houston, Texas, USA; ^3^ Department of Epigenetics and Molecular Carcinogenesis, The University of Texas MD Anderson Cancer Center, Houston, Texas, USA; ^4^ Sylvester Comprehensive Cancer Center, University of Miami, Miami, Florida, USA; ^5^ Department of Radiation Oncology, National Cheng Kung University, National Cheng Kung University Hospital, College of Medicine, Tainan, Taiwan

**Keywords:** cisplatin, arginine-starvation, DEC1-HIF-1α-c-Myc axis, ASS1, DNA methylation

## Abstract

Many human tumors require extracellular arginine (Arg) for growth because the key enzyme for *de novo* biosynthesis of Arg, argininosuccinate synthetase 1 (ASS1), is silenced. These tumors are sensitive to Arg-starvation therapy using pegylated arginine deiminase (ADI-PEG20) which digests extracellular Arg. Many previous studies reported that ASS1 silencing is due to epigenetic inactivation of ASS1 expression by DNA methylation, and that the demethylation agent 5-aza-deoxycytidine (Aza-dC) can induce ASS1 expression. Moreover, it was reported that cisplatin suppresses ASS1 expression through *ASS1* promoter methylation, leading to synthetic lethality to ADI-PEG20 treatment. We report here that cisplatin supppresses ASS1 expression is due to upregulation of HIF-1α and downregulation of c-Myc, which function as negative and positive regulators of ASS1 expression, respectively, by reciprocal bindings to the *ASS1* promoter. In contrast, we found that Aza-dC induces ASS1 expression by downregulation of HIF-1α but upregulation of c-Myc. We further demonstrated that the clock protein DEC1 is the master regulator of HIF-1α and c-Myc that regulate ASS1. cDDP upregulates DEC1, whereas Aza-dC suppresses its expression. Using two proteasomal inhibitors bortezomib and carfilzomib which induce HIF-1α accumulation, we further demonstrated that HIF-1α is involved in ASS1 silencing for the maintenance of Arg auxotrophy for targeted Arg-starvation therapy.

## INTRODUCTION

Many human malignancies including malignant melanoma, hepatocellular carcinoma, prostate cancers, mesothelioma, small cell lung cancer, and breast cancers, do not produce sustainable amounts of arginine (Arg), because the rate-limiting enzyme for biosynthesis of Arg, argininosuccinate synthetase 1 (ASS1), is silenced [1]. These tumors require extracellular Arg in the circulation for survival [1–3]. Arg-starvation therapy using Arg-degrading recombinant proteins, such as pegylated arginine deiminase (ADI-PEG20) which digests Arg into citrulline and ammonia [4] or human arginase 1 which digests Arg into ornithine and urea [5], have been in various stages of clinical evaluations for targeting Arg-auxotrophic tumors [3].

Induction of ASS1 expression has been associated with resistance to ADI-PEG20 treatment [4]. Many reports have shown that ASS1-silencing in Arg-auxotrophic tumors is epigenetically regulated. Nicholson et al [6] reported that aberrant methylation in the *ASS1* promoter correlated with transcriptional silencing of ASS1 in ovarian cancer cells. These authors also showed that epigenetic inactivation of ASS1 is associated with selective resistance to platinum (Pt)-based treatment in cultured cells and in primary ovarian carcinomas. Epigenetic DNA methylation in ASS1-silencing was also reported in nasopharyngeal carcinoma [7], malignant mesothelioma [8], glioblastoma [9], bladder cancers [10], myxofibrosarcomas [11], and lymphoma [12]. Some reports have found that ASS1 silencing can be reversed using the DNA-demethylating agent, 5-Aza-2′-deoxycytidine (Aza-dC) [9, 11], resulting in resistance to ADI-PEG20 treatments. Moreover, a correlation between reduced ASS1 protein levels and the *ASS1* promoter methylation and resistance to cisplatin in hepatocellular carcinoma cell lines was reported [13].

We previously demonstrated that ASS1 silencing is controlled by the transcriptional repressor HIF-1α, which binds the E-box at the *ASS1* promoter. Arg-starvation induces rapid downregulation of HIF-1α and upregulation of another E-box-binding factor c-Myc. De-repression of ASS1 from HIF-1α binding allows c-Myc to activate ASS1 expression [14]. We further demonstrated that upregulation of c-Myc by Arg starvation follows the signal transduction mechanism Ras→PI3K/Akt/ERK→GSK3β, where ERK and GSK3β phosphorylate c-Myc, resulting in c-Myc accumulation by suppressing proteasomal degradation [15]. Recently, we demonstrated that an ROS-related mechanism involving activation of ligand Gas6-dependent-Axl receptor tyrosine kinase (RTK) signal is the sensor of the Arg-activated Ras-transduction pathway in the regulation of ASS1/Arg homeostasis [16].

We report here that suppression of ASS1 expression by cDDP involves elevated expression of HIF-1α and reduced expression of c-Myc, a mechanism opposite to the induction of ASS1 by ADI-PEG20. In contrast, induction of ASS1 by Aza-dC follows the mechanism similar to that for ADI-PEG20. We further demonstrated that another E-box-binding transcription regulator, differentiated embryonic chondrocyte 1 (*DEC1*) (also known as BHLHE40 for basic-helix-loop-helix family member E40, or *Stra13* for stimulated with retinoic acid 13 in mouse and *sharp2* for enhancer of split and hairy related protein 2 in rat), is the master regulator of HIF-1α and c-Myc. These results revealed a novel mechanism of cDDP-induced ASS1 suppression by the transcriptional control of DEC1/c-Myc/ASS1 axis, leading to increased sensitivity to Arg-starvation treatment.

## RESULTS

### cDDP-resistant cells exhibit reduced ASS1 expression and are preferentially sensitive to ADI-PEG20

**Figure 1:**
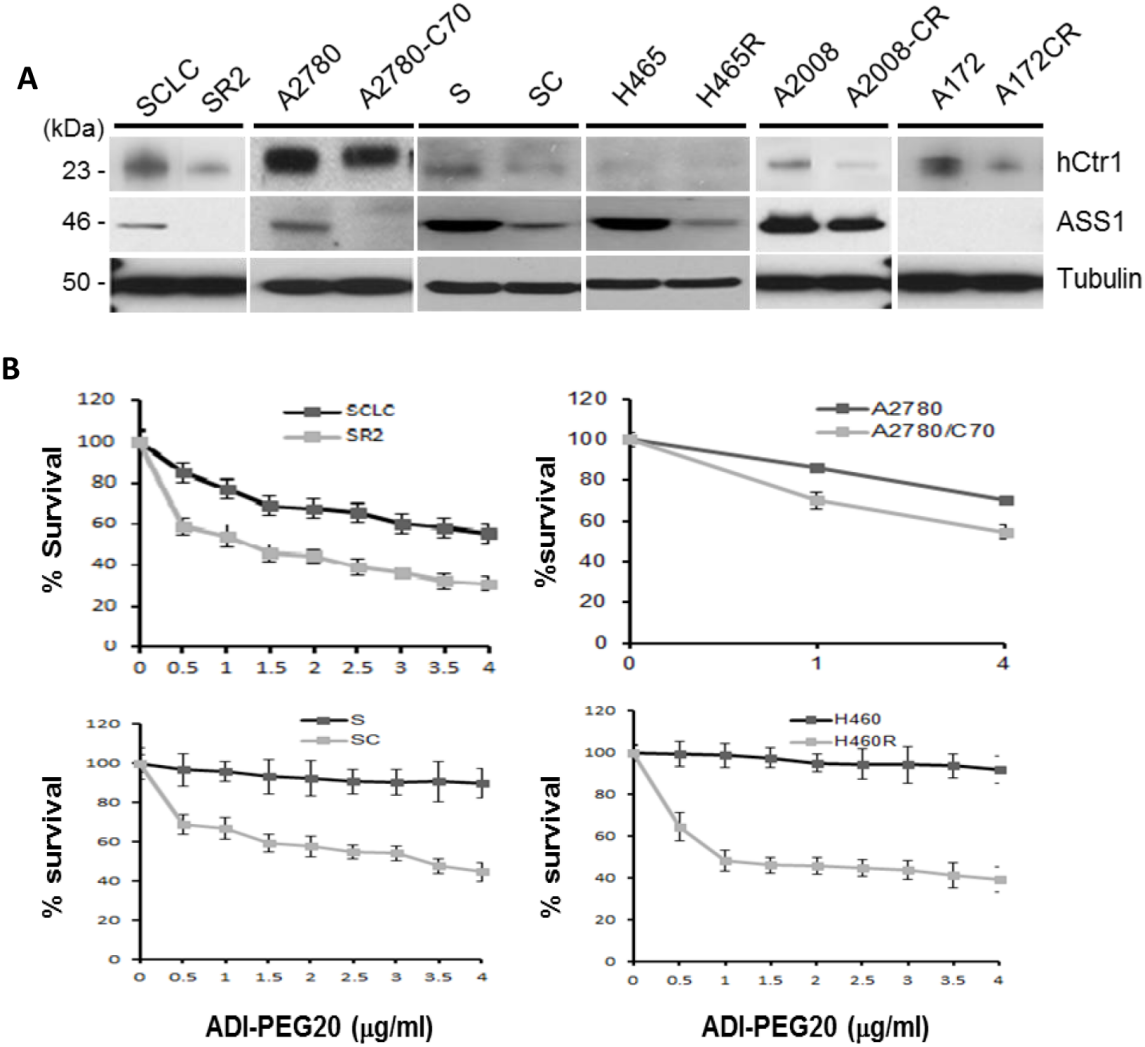
Expression of hCtr1 and ASS1 in cDDP-sensitive and cDDP-resistant cell lines and their differential sensitivities to ADI-PEG20 **A.** Immunoblots showing the expression of hCtr1 and ASS1 in six pairs of cDDP-sensitive (left side) vs cDDP-resistant (right) cell lines. **B.** Concentration-dependent sensitivity to ADI-PEG20 of four randomly chosen pairs of cDDP-resistant *vs* cDDP-sensitive cell lines to ADI-PEG20 for 24 hr as determined by the SRB assay.

We randomly chose six pairs of cDDP-sensitive vs cDDP-resistant cell lines obtained from different laboratories (Figure 1A). These cDDP-resistant cell lines were originally established using continuous exposure to increasing concentrations of cDDP, except A172CR which was established using on-and-off schedule of cDDP treatments for 6 months [17]. All the cDDP-resistant cell lines display reduced levels of the high-affinity copper transporter 1 (hCtr1) as compared with their respective cDDP-sensitive cell lines by Western blotting (Figure 1A). hCtr1 is a cDDP import transporter [18]. These results demonstrate that these cDDP-resistant variants are transport-defective.

ASS1 levels in all the cDDP-resistant cell lines were lower than those in their corresponding cDDP-sensitive counterparts, except the A172-A172CP pair which has undetectable levels of ASS1. These results are consistent with previous findings that reduced ASS1 expression is frequently associated with cDDP resistance [6, 13]. Consequently, cDDP-resistant cell lines were more sensitive than their corresponding sensitive counterparts to the killing by ADI-PEG20 (Figure 1B).

### Suppression of ASS1 by cDDP is due to upregulation of HIF-1α and downregulation of c-Myc

**Figure 2:**
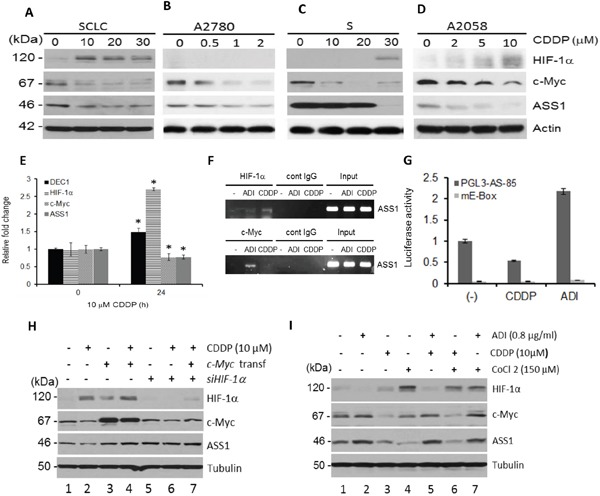
Regulation of HIF-1α, c-Myc, and ASS1 by cDDP **A–D.** Immunoblots showing cDDP concentration-dependent upregulation of HIF-1α and downregulation of c-Myc and ASS1 in four cDDP-sensitive cell lines for 24 hr. **E.** qRT-PER assays of relative mRNA expression levels in SCLC cells treated with 10 μM cDDP for 24 hr. **F.** ChIP assay showing binding of HIF-1α, but not c-Myc, to the *ASS1* promoter in cDDP-treated SCLC cells (10 μM for 24 hr). ADI-treated cells (0.5 μg/ml, 24 hr) were used as references. **G.** Transient transfection of reporter plasmid pGL3-AS-85 which contains wild-type E-box in the ASS1 promoter and mutant E-box containing plasmid (mE- box) in SCLC cells treated with cDDP (10 μM for 24 hr). **H.** Effects of c-Myc-overexpression by transfection, or HIF-1α downregulation by siRNA, on regulation of ASS1 expression in SCLC cells treated with cDDP (10 μM for 24 hr). **I.** Effects of CoCl_2_ on HIF-1α, c-Myc, and ASS1 expression in SCLC cells treated with cDDP alone, ADI-PEG20 alone, or in combination for 24 hr.

We previously reported that ADI-PEG20-induced ASS1 expression is negatively regulated by HIF-1α but positively regulated by c-Myc in cells with low ASS1 expression [14, 15]. To investigate whether the HIF-1α/c-Myc axis is also involved in the regulation of ASS1 by cDDP, we randomly chose four drug-sensitive cell lines, SCLC, A2780, S, and A2058 cells, and treated them with different concentrations of cDDP for 24 hr. Western blots show that HIF-1α expression levels were increased in a cDDP concentration-dependent manner in all these cell lines, except A2780 cells which express undetectable HIF-1α, whereas levels of c-Myc and ASS1 expression were reduced as cDDP concentrations were increased (Figure 2A–2D). Using quantitative real time-polymerase chain reaction (qRT-PCR) assay, we demonstrated that HIF-1α and DEC1 mRNA (see below) levels were increased but c-Myc and ASS1 mRNA were reduced in SCLC cells treated with cDDP (Figure 2E), consistent with the protein expression patterns (Figure 2A). We next used the ChIP assay to confirm that negative regulation of ASS1 by cDDP is mediated by increased *ASS1* promoter-binding of HIF-1α but not c-Myc. We used ADI-PEG20 treatment which induces c-Myc, but not HIF-1α binding for comparison (Figure 2F). cDDP transcriptionally suppresses ASS1 expression was also demonstrated by transient transfection assay using pGL3-AS-85-luc reporter, which contains 85 nucleotides of *ASS1* promoter harboring the wild-type E-box sequence, but not the mutant E-box (mE-Box) sequence (Figure 2G). These results demonstrate that HIF-1α/c-Myc axis is also involved in ASS1 suppression

To support the negative role of HIF-1α and positive role of c-Myc in cDDP-induced ASS1 downregulation, we overexpress c-Myc by transfection with recombinant and found it upregulates ASS1 expression with or without cDDP treatment (Figure 2H, lanes 3 & 4 vs lane 1). Knockdown HIF-1α using the siRNA increased ASS1 expression (Figure 2H, lanes 5 and 6). In these experiments, the magnitudes of ASS1 and c-Myc regulation were not high (within ± 1.8-fold each) by ImageJ densitometer scanning. The reason for the low magnitudes of induction can be explained by homeostatically mutual regulation between ASS1 and c-Myc. We recently demonstrated that while elevated c-Myc induced by ADI-PEG20 upregulates ASS1, but elevated ASS1 feedback-inhibits c-Myc expression. Moreover, c-Myc expression is homeostatically self-regulated [16]. Co-treatment with cDDP and ADI-PEG20 abrogated the negative effect of HIF-1α in the regulation of ASS1 (Figure 2I, lanes 3 and 5). CoCl_2_ which causes HIF-1α accumulation by inhibiting the prolyl hydroxylase (PHD) activity for the proteasomal degradation of HIF-1α suppresses ASS1 expression (Figure 2I, lane 4) and further suppress ASS1 expression when combined with cDDP (Figure 2l, lanes 3 *vs* 6). ADI-PEG20 alone downregulates HIF-1α but upregulates c-Myc and ASS1 (Figure 2l, lanes 2 *vs* 1), and together with CoCl_2_ reduces HIF-1α but increases c-Myc and ASS1 expression (Figure 2I, lanes 4 *vs* 7). These results, collectively, demonstrate that cDDP-suppressed ASS1 expression is negatively regulated by HIF-1α and positively regulated by c-Myc.

### The DEC1 transcriptional regulator is the master regulator of HIF-1α and c-Myc in cDDP-regulated ASS1 expression

**Figure 3:**
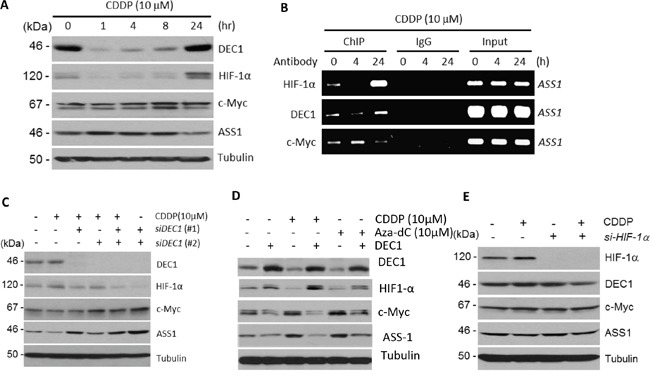
Regulation of DEC1, HIF-1α, c-Myc, and ASS1 by cDDP **A.** Time-dependent protein expression levels as indicated in cDDP-treated SCLC cells. **B.** ChIP assay showing time-dependent bindings of DEC1, HIF-1α and c-Myc to the *ASS1* promoter. **C.** Effects of DEC1 knockdown by two siRNAs on the expression of HIF-1α, c-Myc, and ASS1 in SCLC cells treated with or without cDDP for 24 hr. **D.** Effects of overexpression of DEC1 by transfection on HIF-1α, c-Myc and ASS1 expression. Transfection were performed for 24 hr followed by treatment with cDDP or Aza-dC for 5 hr at the concentrations indicated. **E.** Effect of HIF-1α knockdown by siRNA on the expression DEC1, c-Myc, and ASS1. SCLC cells were treated with cDDP (10 μM) with or without prior si-HIF-1α transfection for 24 hr as indicated.

We next investigated how HIF-1α and c-Myc are regulated by cDDP. Both c-Myc [16] and HIF-1α [19] promoters contain E-box sequences, suggesting that another E-box-interacting transcriptional regulator may be the master regulator of HIF-1α and c-Myc in response to cDDP. Among the many E-box-interacting transcriptional regulators described in the literature, we focused on DEC1 which has been implicated to cross-talk with HIF-1α [20] and c-Myc [21]. Treating SCLC cells with cDDP induces rapid downregulation of DEC1 within one hr, but levels of DEC1 gradually increased thereafter. At 24 hr, levels of DEC1 were higher than that in untreated control. The expression of HIF-1α positively followed DEC1 whereas ASS1 inversely followed DEC1 in a temporal manner, although the magnitudes of increment ASS1 were not as high as those for DEC1 and HIF-1α (Figure 3A). Time-course changes of c-Myc levels somewhat follow that of ASS1. Importantly, temporally dependent rise-and-fall of DEC1, HIF-1α and c-Myc in cDDP treatments were reflected by their bindings to the *ASS1* promoter as shown by ChIP (Figure 3B). Particularly, the observation that cDDP also induces DEC1 binding to the ASS1 promoter in a time-dependent manner suggests that DEC1 may also regulate ASS1 expression. We note that in Figure 3A (and some following figures), c-Myc appears as a doublet in the Western blots. These doublets may be due to post-translational modification of c-Myc, such as acetylation (see below).

That DEC1 positively regulates HIF-1α but negatively regulates c-Myc were demonstrated using siRNA knockdown and *DEC1* transfection approaches. Knockdown DEC1 by two DEC1 siRNAs suppressed cDDP-induced HIF-1α downregulation, but enhanced c-Myc and ASS1 expression (Figure 3C). Overexpression of DEC1 by transfection increases HIF-1α but suppressed c-Myc and ASS1 expression (Figure 3D). While DEC1 strongly regulates HIF-1α, however, it appears that HIF-1α may not feedback-regulate DEC1, because knockdown HIF-1α shows only marginal downregulation of DEC1 (Figure 3E). These results suggest that cDDP can positively and negatively regulate DEC1 in a time-dependent manner, and that DEC1 is an upstream transcription factor regulating HIF-1α and c-Myc; both then regulate ASS1 in a time-dependent manner. Alternatively, cDDP may transiently suppress DEC1 expression and then gradually recovered.

### Modulations of DEC1 and HIF-1α/c-Myc/ASS1 by histone deacetylases

**Figure 4:**
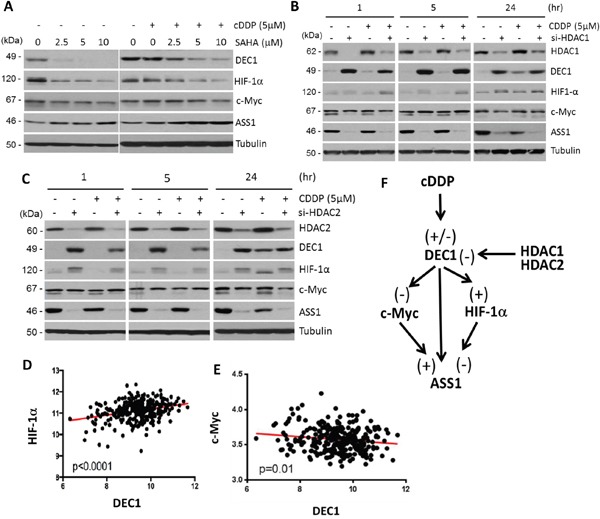
Effects of HDACi on expression of DEC1, HIF-1α, c-Myc, and ASS1 **A.** SCLC cells were treated with different concentrations of SAHA alone (left panel) or in combination with cDDP (right) for 5 hr. Protein expression were determined. **B** and **C.** Effects of HDAC1 or HDAC2 knockdown on the expression of DEC1 and other proteins as indicated. **D.** Positive correlation between HIF-1α and DEC1 expression in the ovarian cancer database. **E.** Negative correlation between c-Myc and DEC1 expression in the ovarian cancer database. **F.** Schematic diagram showing regulation of DEC1 and its downstream targets (c-Myc, HIF-1α, and ASS1) by cDDP and HDAC1 and HDAC2. (−) and (+) denote negative and positive regulations, respectively.

Previous studies have demonstrated that DEC1 expression is controlled by histone deacetylase inhibitors (HDACi) [22, 23]. To investigate whether cDDP-regulated DEC1 also involves histone deacetylation, we used the HDACi, suberoylanilide hydroxamic acid (SAHA, vorinostat) which inhibits all 11 members in human classes I and II HDACs [24]. Treating SCLC cells with SAHA at concentration ranging 2.5 – 10 μM for 5 hr reduced the expression of DEC1. Expression of HIF-1α was coordinately reduced, but levels of ASS1 expression were increased, and no apparent change of c-Myc was observed (Figure 4A, left panel). These results are consistent with the findings that DEC1 positively and negatively regulate HIF-1α and c-Myc, respectively. We also found that cDDP attenuated the suppressive effects of DEC1 and HIF-1α induced by SAHA (Figure 4A, right panel). We did not observe substantially further increased ASS1 expression by addition of 5 μM and 10 μM SAHA in the cDDP (5 μM)-treated cells. This may be explained that ASS1 is homeostatically autoregulated as mentioned [16].

We next used siRNA to knockdown specific HDACs. Among the 11 members in the Classes I and II HDACs, HDAC1, 2, and 3 are constitutively nuclear and play important roles in transcriptional regulation of a broad spectrum of genes [25]. Furthermore, previous studies demonstrated that DEC1 physically interacts with HDAC1 [26] and HDAC2 [27]. To investigate whether these three HDACs may regulate DEC1 expression under cDDP challenge, SCLC cells were transfected with siRNA for HDAC1 (Figure 4B) or HDAC2 (Figure 4C) for 48 hr or left untreated. Cells were then treated with or without cDDP (5 μM) for 1, 5, and 24 hr. We found that cDDP treatment slightly enhanced HDAC1 and HDAC2 expression. Interestingly, knockdown HDAC1 or HDAC2 resulted in increased DEC1 expression accompanied with increased HIF-1α but decreased c-Myc and ASS1 expression. Knockdown HDAC3 did not have such effects (data not shown), demonstrating isoform-specificity in the regulation of DEC1 by HDACs. These observations are consistent with the results that DEC1 positively regulates HIF-1α but negatively regulates c-Myc and ASS1. The discrepancies between these results and those from using SAHA will be discussed below. Both HIF-1α [28] (and references therein) and c-Myc [29] are subject to posttranslational modifications such as acetylations at multiple sites. Acetylated HIF-1α and c-Myc may have slower mobilities in SDS-PAGE than their unacetylated counterparts, resulting in the appearance of doublets in Western blots. This may explain that knockdown of HDAC1 (Figure 4B) or HDAC2 (Figure 4C) by siRNA results in enrichments of the acetylated forms (upper bands).

### Correlations of DEC1, HIF-1α, and c-Myc expression in tumor specimens

To determine the relationships among DEC1, HIF-1α and c-Myc expression in tumor specimens, we analyzed a microarray gene expression profiling dataset derived from 285 serous and endometrioid tumors of ovary, peritoneum, and fallopian tube using affynetrix U133 Plus 2 arrays [30] (NCBI GSE9891). Strongly positive correlation between DEC1 and HIF-1α expression (*p*<0.001) (Figure 4D), but negative correlation between DEC1 and c-Myc (*p*=0.01) (Figure 4E), were observed.

These results, taken together, support the transcriptional network of ASS1 expression by cDDP as depicted in Figure 4F. cDDP can positively (+) or negatively (−) regulate DEC1 expression in a time-dependent manner. DEC1 then positively regulates HIF-1α but negatively regulates c-Myc, both of which in turn regulates ASS1 expression in opposite ways. Moreover, both HDAC1/HDAC2 are strong negative regulators of DEC1.

### Aza-dC induced-ASS1 expression is also regulated by the DEC1/HIF-1α/c-Myc axis

**Figure 5:**
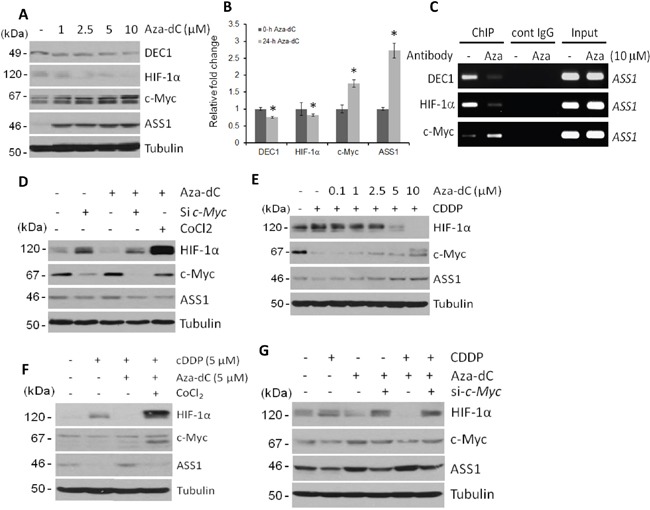
Regulation of DEC1, HIF-1α, c-Myc and ASS1 by Aza-dC **A.** Protein expression in SCLC cells treated with different concentrations of Aza-dC for 24 hr. **B.** qRT-PCR assays of relative mRNA levels in SCLC cells treated with 10 μM of Aza-dC for 24 hr. (* refer to p<0.05 but the student's *t* test). **C.** ChIP assays of DEC1, HIF-1α, and c-Myc bindings to the *ASS1* promoter in Aza-dC-treated SCLC cells for 24 hr. **D.** Effects of c-Myc siRNA and CoCl_2_ on protein expression in SCLC cells treated with Aza-dC (10 μM for 24 hr). **E.** Concentration-dependent rescue of cDDP-induced ASS1 suppression by Aza-dC in SCLC cells treated with cDDP (10 μM for 24 hr). **F.** Effects of CoCl_2_ on the regulation of HIF-1α, c-Myc and ASS1 by cDDP and Aza-dC for in SCLC cells treated for 24 hr at concentrations as indicated. **G.** Effects of c-Myc siRNA on the regulation of HIF-1α, c-Myc, and ASS1 treated with cDDP (5 μM) with or without Aza-dC treatment (10 μM for 24 hr).

Aza-dC-induced ASS1 expression was implicated by the mechanism of DNA demethylation [6, 7, 9–13, 31]. However, we found a concentration-dependent Aza-dC-induced downregulation of DEC1 and HIF-1α but upregulation of c-Myc and ASS1 (Figure 5A). These protein expression patterns are correlated with the mRNA patterns as determined by qRT-PCR (Figure 5B). Moreover, downregulation of DEC1 and HIF-1α but upregulation of c-Myc by Aza-dC was correlated with their bindings to the *ASS1* promoter (Figure 5C). The negative role of HIF-1α in Aza-dC-induced ASS1 expression was confirmed using inhibitor to HIF-1α degradation, CoCl_2_, and the positive role of c-Myc was demonstrated by using c-Myc siRNA (Figure 5D). Moreover, ectopic expression of DEC1 enhances HIF-1α expression with downregulation of c-Myc, resulting in suppression of Aza-dC-induced ASS1 expression (Figure 3D). We further demonstrated that Aza-dC was able to reverse the inhibitory effect of ASS1 expression by cDDP (Figure 5E), and that the reversal was abrogated by CoCl_2_ (Figure 5F) and by c-Myc siRNA treatments (Figure 5G). These results, taken together, demonstrated that Aza-dC-induced ASS1 expression is transcriptionally regulated via DEC1/HIF-1α/c-Myc signaling.

### The transcriptional start site and E-box sequences of *ASS1* are not methylated

We used a bisulfite-pyrosequencing approach to investigate the methylation status of several CpG islands located at the E-box and the transcriptional start site which is located in exon 1 of the *ASS1* gene. We found that these sites are virtually unmethylated in SCLC cells and that cDDP treatment did not alter the methylation states. No alteration of methylation status in response to cDDP was also observed at the c-Myc promoters (Supplementary Table 1). These results demonstrate that DNA methylation at these areas did not play a significant role in silencing ASS1 expression.

### Enhanced ADI-PEG20's cell-killing capacity by proteasome inhibitors for HIF-1α

**Figure 6:**
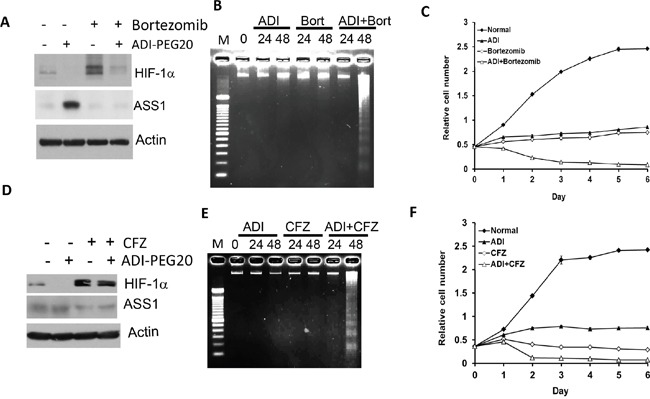
Inhibition of HIF-1α degradation by bortezomib or carfizomib increases sensitivity of A2058 cells to ADI-PEG20 treatment **A.** Western blots of A2058 cells treated with bortezomib (5 nM), ADI-PEG20 (1 μg/ml) and in combination for 24 hr, **B.** enhancement of apoptosis by ADI-PEG20 and bortezomib cotreatment, **C.** enhanced sensitivity to cell killing by cotreatment with ADI-PEG20 and bortezomib. **D–F.** Similar experiments corresponding to those presented in (A – C) except using carfilzomib (20 nM) and ADI-PEG20 (0.3 μg/ml), instead of bortezomib, and treatment time was 8 hr.

To test the clinical implications of HIF-1α in ADI-PEG20 sensitivity, we used two FDA-approved HIF-1α degradation inhibitors, bortezomib and carfilzomib (CFZ). Both are proteasomal inhibitors for treating multiple myeloma [32]. We used A2058 melanoma cells because they are auxotrophic for arginine [14, 15, 33]. Treating A2058 cells with bortezomib alone for 24 hr induces significant accumulation of HIF-1α, and addition of ADI-PEG20 reduces HIF-1α accumulation induced by bortezomib. Bortezomib alone or in combination with ADI-PEG20 suppress ASS1 (Figure 6A).

We used DNA fragmentation assay to determine drug-induced apoptosis. While no apparent apoptosis was observed in cells treated with bortezomib or ADI-PEG20 alone or both for 24 hr, apoptosis was evident only in the combination treatment for 48 hr (Figure 6B). These results demonstrate that ADI-PEG20 and bortezomib have synergistic effect on apoptosis induction, and the induction is a delay mechanism. Enhanced ADI-PEG20's cytotoxicity by the combination of bortezomib and ADI-PEG20 is also observed using cell-killing assay (Figure 6C). Similar effect was seen by carfilzomib (Figures 6D–6F). We conclude that proteasomal antitumor agents can enhance ADI-PEG20 cell killing via ASS1 suppression.

## DISCUSSION

Platinum-based antitumor agents have been the mainstay of cancer chemotherapy for more than three decades. Drug resistance is an important factor for the treatment failure. The findings in this communication and in previous publications [6, 13] demonstrating that cDDP-resistant variants exhibit reduced expression of ASS1, render them vulnerable to Arg-starvation treatment by ADI-PEG20. These findings highlight the potential of Arg-starvation strategy in overcoming platinum-based drugs.

**Figure 7:**
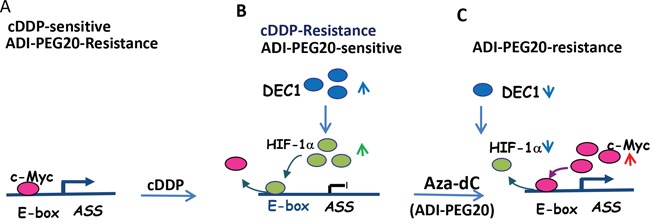
Schematic depicting regulation of ASS1 expression by various transcriptional regulators treated with cDDP and Aza-dC **A.** Before treatment, expression of ASS1 in normal cells is controlled by c-Myc, which interacts with the E-box located at the *ASS1* promoter. **B.** cDDP induces DEC1 expression which positively regulates HIF-1α and negatively regulates c-Myc, resulting in switching of HIF-1α and c-bindings to the ASS1 promoter and ASS1 silencing. **C.** Aza-dC reverses the effects of cDDP by down-regulation of DEC1 and HIF-1α but upregulation of c-Myc, which re-occupies the E-box and activates ASS1 expression.

Mechanisms for ASS1-silencing induced by cDDP are controversial. Previous studies have concluded that reduced ASS1 expression is mediated by DNA methylation. However, we demonstrated here that silencing of ASS1 expression by cDDP is primarily due to transcriptional suppression by HIF-1α (Figures 7A and B). This suppression can be rescued by DNA demethylating agent Aza-dC and by ADI-PEG20 (Figure 7C). These agents induces rapid HIF-1α degradation, allowing the positive transcriptional factor c-Myc to activate ASS1 expression (Figure 7C). By their interchanging occupancies at the promoter, c-Myc and HIF-1α control the on-and-off of ASS1 expression. This model is consistent with our previous observations [14, 15].

Another important finding in this communication is the discovery that DEC1 is the master regulator of HIF-1α and c-Myc for their regulation of ASS1. These have been demonstrated for cDDP-induced ASS1 repression and in Aza-dC-induced reactivation (Figure 7). In both situations, DEC1 positively regulates HIF-1α but negatively regulates c-Myc. The opposite functions of DEC1 in different promoter contexts have been reported previously [21, 34, 35]. DEC1 can also induce protein stabilization by interference of ubiquitin-mediated protein degradation [36]. Nonetheless, the precise mechanism(s) on how cDDP/Aza-dC regulate DEC1 and its downstream targets (HIF-1α and c-Myc) require critical investigations.

Of particularly intriguing is the time-dependent regulation of DEC1 by cDDP, with rapid initial downregulation followed by a gradual increase to a level higher than that in the initial time point 24 hr after the treatment. This results in elevated HIF-1α expression to suppress ASS1 for synthetic lethality by ADI-PEG20. Previous studies have demonstrated that expression of DEC1 can be regulated by many environmental stimuli, including nutritional feeding [37], growth factor [38], and genotoxic agents [39, 40]. In mammalian cells, DEC1 functions as a repressor for the circadian transcriptional regulators CLOCK/BMAL through promoter E-box binding [20]. DEC1 itself also shows remarkable circadian rhythmic expression in response to light-pulse [41, 42]. In addition to the rhythmic expression in the day-light cycle, its expression can be dynamically regulated by the vitamin D receptor (VDR) ligand 1α,25(OH)_2_D3 which follows a stair case style fluctuation in interval of less than 60 min [23]. These findings may explain our current results showing that cDDP-induced DEC1 fluctuates within 24 hr. Given that DEC1 is a clock protein and its expression levels are in periodicity, it may be of importance to investigate whether regulation of ASS1 by cDDP is also rhythmically regulated. This research may have important implications in ADI-PEG20 therapy.

Regulation of DEC1 is also controlled at the epigenetic level. Previous study demonstrated that the dynamic regulation of DEC1 by the VDR ligand is rapidly downregulated by the HDACi, trichostatin A [23]. Consistent with this finding, we found that SAHA also downregulates DEC1. However, targeted inhibitions of HDAC1 and HDAC2 (but not HDAC3) using an siRNA approach resulted in an opposite effect, *i.e*, enhanced DEC1 expression. These results raise the complexity of DEC1 regulation by HDACs, and demonstrate that caution is needed in investigations using HDACi. While the mechanisms of these discrepancies are currently not known, but explanations maybe offered: (*i*) SAHA has a broad inhibitory spectrum of HDAC by binding to the active sites of multiple HDACs and acts as a chelator for Zn ions at the active site [43]. Different gene expression patterns by the pan-HDACi SAHA and by the more isoform-specific HDACi such as MRLB22 have been reported. (*ii*) In this study, we treated cells with SAHA for 5 hr, whereas knockdown of HDAC1 and HDAC2 required approximately 24 hr to see the effects. Given the fact that DEC1 expression is temporally regulated by cDDP, it is possible that regulation of DEC1 by HDACi may be also time-dependent. Indeed, temporal fluctuations in gene regulation by SAHA (and other HDACi) was reported previously [44]. Future studies are required to elucidate the mechanisms involved in the differential regulation of DEC1 by SAHA (and other HDACi) and HDAC siRNA. Nonetheless, it is important to note that by using HDACi (SAHA) and HDAC siRNA, our results both support the notion that DEC1 is the upstream regulator of the HIF-1α/c-Myc axis in ASS1. Nonetheless, further studies are required to delineate the precide mechanisms underlying how histone modifications are involved in this axis.

Finally, our results demonstrating that HIF-1α plays a suppressive role in controlling ASS1 expression reinforce the silencing mechanism of ASS1 as we have previously reported [14–16]. cDDP-induced HIF-1α expression resulting in sensization to ADI-PEG20 treatment supports the current ongoing clinical trials using cDDP and ADI-PEG20 in treating multiple tumor types (NCT0166518) [3]. The observations that bortezomib and carfilzomib can also potentiate the cell-killing efficacy of ADI-PEG20 through enhanced HIF-1α expression may provide another avenue for combination therapy in improving the treatment efficacy of Arg-starvation treatment.

## MATERIALS AND METHODS

### Reagents, recombinant DNA, antibodies, and cell culture

Cisplatin, Aza-dC, CoCl_2_ and sulforhodamine B (SRB) were obtained from Sigma-Aldridge. ADI-PEG20 (specific activity, 5-10 IU/mg) and mouse monocloncal anti-ASS1 antibody were obtained from Polaris Pharmacologies, Inc (San Diego, CA). Additional antibodies were obtained from the following commercial sources: rabbit anti–c-Myc (N262) and mouse anti–c-Myc (C33) from Santa Cruz Biotechnology, mouse anti-HIF1α (cat no. 610958) from BD Bioscience, rabbit anti-DEC1 antibody from Bethyl Lab.

The siRNA for HIF-1α (5′-GAUUAACUCAG UUUGAACUdTdT) was synthesized by Sigma, c-Myc siRNA was purchased from Cell Signaling, and control siRNA were from Santa Cruz Biotech. DEC1 siRNA (5'- GCAAGGAGACCUACAAAUUdTdT); HDAC1 siRNA (#1, 5′-CAGCGACUGUUUGAGAACCdTdT, and #2, 5′-UCCGUAAUGUUGCUCGAUGdTdT) and HDAC2 siRNA (5' AAGCAUCAGGAUUCUGUUAdTdT) were synthesized by Sigma. Recombinant DEC1 cDN (RC213429) was obtained from Origene. pCGN-HA-c-Myc was generously provided by Dr. William P. Tansey, Ruttenberg Cancer Center, New York, NY) [45]. The siRNA and plasmid DNA were transfected using lipofectamine 2000 (Invitrogen) according to the manufacturer's instruction.

Small-cell lung cancer cell (SCLC vs SR2) [46] and non-small cell lung cancer (NSCLC) (NCI-H460 vs H465CR, and S vs SC) were obtained from N. Savaraj, ovarian cancer (A2780 vs A2780CP70 [47]) were from Thomas C. Hamilton (Fox Chase Cancer Center, Philadelphia, PA), and human ovarian adenocarcinoma (A2008 vs A2008CR) [48] were obtained from Zahid Siddik (MD Anderson Cancer Center) and glioblastoma (A172 vs A172CR) were from Akira Gomi [17] (Jichi Medical School, Tochigi, Japan). Melanoma cell line A2058 was obtained from American Type Culture Collection. Descriptions of these cell lines were given in the references cited, except NSCLC which were obtained from human tumor biopsies. These cell lines have been kept in liquid N2 for various lengths of time ranging from 3 to 7 years before use. cDDP-resistant phenotypes were not changed at the time this study was initiated as characterized by the reduced expression of hCtr1. Cells were maintained in DMEM containing 10% fetal bovine serum at 5% CO_2_ at 37°C. cDDP-resistant cell lines were maintained in the regular medium containing 0.5 μg/ml cDDP.

### Quantative real time-polymerase chain reaction (qRT-PCR)

Total RNA was isolated with trizole reagent (Invitrogen) following manufacturer's instruction. The cDNA was synthesized from 1 μg of total RNA using a Super Script II system (Invitrogen). The synthesized cDNA was subjected to standard real-time PCR using following oligonucleotide primers, *c-Myc*: forward (f), 5′-CCTACCCTCTCAACGACAGC-3′ and reverse (r), 5′-ACTCTGACCTTTTGCCAGGA; *ASS1*, (f) 5′-AGGCACCATCCTTTACCATG and (r) 5′-CTGCA CTTTCCCTTCCACTC; *HIF-1α*, (f) 5′-TGCATCTCC ATCTCCTACCC, (r) 5′-CGCTTTCTCTGAGCATTCTG; *DEC1*, (f) CTTCAGTATCTGGCCAAGCA, (r) TGAAG TCCATCACTTTGGGA; β-actin, (f): 5′-GAGGCCCAG AGCAAGAGAG and (r) 5′-AGAGGCGTACAGG GATAGCA.

### Chromatin immunoprecipitation assay

ChIP assay was carried out with the ChIP assay kit (Upstate Biotechnology) following manufacturer's instruction. A GC-rich PCR system (Roche) was used using *ASS1* promoter–specific primers: (f) 5′-TGAGTTACATGGGTCGCAGCCACTG and (r) 5′-GCCCATCCCAGGTTATAAGCACAGG). Assay of the *ASS1* promoter DNA were performed as described previously [14, 15].

### Methylation analysis with pyrosequencing

Five hundred ng of genomic DNA was treated with sodium bisulfite using the EZ DNA Methylation-Gold Kit (Zymo Research, Irvine, CA) according to the manufacturer's protocol. The samples were eluted in 40 μl of M-Elution Buffer, and 2 μl (equivalent to 25 ng of bisulfite-modified DNA) were used for each PCR reaction. Both bisulfite conversion and subsequent pyrosequencing analysis were done at the DNA Methylation Analysis Core, The University of Texas M.D. Anderson Cancer Center.

PCR primers for the genomic area proximal to the transcription start site of the genes of ASS1 and c-Myc were designed using the Pyromark Assay Design SW 1.0 software (Qiagen, Hilden, Germany). PCR reactions were performed in a total volume of 15 μl. PCR product was purified with streptavidin-sepharose high-performance beads (GE Healthcare Life Sciences, Piscataway, NJ). Co-denaturation of the biotinylated PCR products and sequencing primer (3.6 pmol/reaction) was conducted following the PSQ96 sample preparation guide. Controls for high methylation (SssI-treated DNA), low methylation (WGA-amplified DNA), partial methylation (equimolar mixture of SssI-treated and WGA-amplified DNA) and no-DNA template were included in each reaction. Sequencing was performed on a PSQ HS 96 system (Biotage AB, Uppsala, Sweden) with the PyroMark Gold Q96 CDT Reagents (Qiagen, Hilden, Germany) according to the manufacturer's instructions. The degree of methylation was calculated using the Pyro-Q CpG 1.0.9v software (Biotage AB, Uppsala, Sweden).

### Other procedures

Procedures of western blotting, apoptosis analysis of DNA fragmentation, and cell growth sensitivity test using sulforhodamine B (SRB) assay were described previously [14, 15].

## SUPPLEMENTRY TABLE


