# Supplementary material for: Cisplatin-induced synthetic lethality to arginine-starvation therapy by transcriptional suppression of ASS1 is regulated by DEC1, HIF-1α, and c-Myc transcription network and is independent of ASS1 promoter DNA methylation
Source: Oncotarget. 2016 Sep 28;7(50):82658–70. doi: 10.18632/oncotarget.12308 (PMC5347722; doi:10.18632/oncotarget.12308)
Supplement: Supplementary file 1 [file oncotarget-07-82658-s001.pdf]

Cisplatin-induced synthetic lethality to arginine-starvation therapy by transcriptional suppression of ASS1 is regulated by DEC1, HIF-1α, and c-Myc transcription network and is independent of *ASS1* promoter DNA methylation

SUPPLEMENTRY TABLE

Supplementary Table S1: Percent of DNA methylation at the selected regions as analyzed by pyrosequencing

Sample ID	ASS1_E-box	ASS1_Exon 1	MYC_Ebox	MYC_P1_Exon1	MYC_P2_Exon 1
SCLC(-)	3.65	3.50	8.39	1.09	0.61
SCLC_cDDP	3.92	3.75	7.85	1.29	0.81
SssI	96.95	96.12	85.10	97.98	97.87
WGA	0.93	1.84	9.76	2.13	1.07

SCLC cells were treated with cDDP (10 μg/ml) for 24 hr (SCLC_cDDP) or untreated (SCLC(-)).
Selected regions include:
ASS1_E-box, E-box at the *ASS1* promoter;
ASS1_Exon1, Exon 1 of *ASS1* locus including the transcription start site;
MYC_Ebox; E-box located at the *c-Myc* promoter;
MYC_P1, romoter 1 at the ASS1 promoter;
MYC_P2; Promoter 2 at the *ASS1* promoter;
SssI, CpG methylase-treated DNA (as a positive control);
WGA, whole genome amplified DNA (as a basal line control).
